# The discrepancy between fire ant recruitment to and performance on rodent carrion

**DOI:** 10.1038/s41598-021-04051-8

**Published:** 2022-01-07

**Authors:** Constance Lin, Aaron M. Tarone, Micky D. Eubanks

**Affiliations:** Department of Entomology, Texas A&M University, Minnie Belle Heep, 2475 TAMU, College Station, TX 77843-2475 USA

**Keywords:** Ecology, Community ecology, Ecosystem ecology, Invasive species

## Abstract

Ants have not been considered important in the process of vertebrate carrion decomposition, but a recent literature review reported over 150 carrion-visiting ant species. Though many ant species have been observed to remove carrion tissue and consume carrion-exuded liquids, the significance of ant recruitment to vertebrate carrion is poorly understood. We conducted a combination of field and laboratory experiments to quantify red imported fire ant recruitment to rodent carrion and determine whether consuming rodent carrion is beneficial to ant colony performance. In the field, 100% of rat carcasses were rapidly colonized by fire ants at high abundances. In our laboratory experiment, the performance of mice-fed fire ant colonies was poor when compared to colonies that were fed mice and insects or insects only. Our results suggest that there is a discrepancy between high levels of fire ant recruitment to vertebrate carrion and the poor colony performance when fed carrion. We hypothesize that fire ants are attracted to vertebrate carrion not because it is a high-quality food, but rather because it hosts large numbers of other invertebrates that can serve as prey for fire ants, potentially showcasing an interesting case of tritrophic interaction in carrion ecology.

## Introduction

Vertebrate carrion decomposition is a crucial step in nutrient cycling in which energy, minerals and nutrients are redistributed back into the ecosystem^1,2^. Understanding the process of decomposition and the role played by various organisms has broad ecological and forensic applications. Insects are amongst the most important facilitators of vertebrate carrion decomposition^3,4^. Flies and beetles (Diptera and Coleoptera) are very well-studied as they constitute most of the vertebrate carrion-visiting insect assemblage. These vertebrate carrion-visiting flies and beetles are primary colonizers and prolific biomass consumers which make them important in decomposition ecology and forensic entomology^5–7^. Though relatively uncommon, other insects are occasionally observed on vertebrate carrion. Which includes wasps^8^, bees^3^, katydids^9^, mantids^7^ and ants^10,11^. Some of these insect species directly ingest vertebrate carrion, other species use vertebrate carrion as shelter or predate invertebrates attracted to carcasses^12,13^.

Although ants (Hymenoptera: Formicidae) are not usually considered as part of the vertebrate carrion insect community, over 150 ant species, representing nine subfamilies, have been reported in or around vertebrate carrion^11^. Their role as vertebrate carrion-visitors remains understudied and our knowledge is limited to isolated reports of ants feeding on carcasses, predating carrion-feeding insects, or both^13–16^. Studies directly addressing the influence of ants on vertebrate carrion decomposition are scarce and provide conflicting conclusions. The skin lesions created by ants could potentially accelerate the decomposition process by causing epidermal ripping during the bloating stage, thereby providing quicker access for other insects to the inside of carcasses^17–19^. In contrast, the presence of ants could exclude insect decomposers from the carcass or predate larval and adult flies; predation of carrion feeding insects has been shown to decelerate decomposition rates^3,20–24^. In addition, Lindgren et al.^23^ documented a behavior exhibited by the red imported fire ant (*Solenopsis invicta*) in which nest-like structures were built directly on the surface of the corpse, leading to an eight-day delay of blow fly colonization. Other studies have investigated the effect of fire ants on invertebrate decomposers and found that the presence of fire ants effectively excluded flies and beetles from mouse carcasses^19,21^.

Although several studies have reported how ants affect vertebrate carrion decomposition either directly by the active removal and damaging of tissues or indirectly through predation, exclusion, and habitat alteration^11^, the mechanisms behind ant attraction to vertebrate carrion remain largely speculative. To our knowledge, no studies have investigated the rate or the magnitude of fire ant recruitment to vertebrate carrion and no study has compared ant recruitment with ant performance when fed vertebrate carrion (preference versus performance). Our current knowledge of ant nutritional requirements is still fragmentary and has mainly focused on the colony-level regulation of protein-carbohydrate intake^25^. The regulation of ants’ protein-lipid intake, which comes primarily from consuming prey or carrion, is still in its infancy^26^. The importance of vertebrate carrion in ants’ diet remains an unanswered question.

The two main objectives of this study are to provide a quantitative estimate of the recruitment of red imported fire ants (*Solenopsis invicta*) to vertebrate carrion (using rodent carrion as a representative), and to investigate why fire ants recruit to vertebrate carrion. The red imported fire ant is an invasive species present at very high densities in the southeastern United States^27^, they are also an opportunistic feeder previously reported on vertebrate carrion^28^, we predicted that rat carcasses would be rapidly colonized in the field. For the second objective, we hypothesized that the performance of fire ant colonies (estimated by brood production, worker mortality/production, reproductive alate production and worker lipid stores) would be enhanced by the addition of vertebrate carrion to their diet. To test these hypotheses, we conducted a field experiment to assess the level of fire ant colonization of vertebrate carrion. We quantified red imported fire ant activity and recruitment at 6, 48 and 72 h after rat carcasses were left in the field to decompose. We also tested whether a diet of vertebrate and/or insect carrion would affect the survival, brood production, production of reproductives, and lipid stores of workers in laboratory-maintained colonies of the red imported fire ant constrained to mice carrion, insect carrion or a mixture of mice and insect carrion diets.

## Results

### Field experiment

A total of 126,181 fire ants were collected from the pitfall traps throughout the 28 replications of the field experiment. All rat carcasses (n = 84) placed in the field were colonized by *Solenopsis invicta.* The number of rat carcasses included in analysis was lower than expected due to scavenging/removal by vertebrates (i.e., raccoons). Several other ant species were collected, including *Brachymyrmex patagonicus, Cyphomyrmex rimosus, Forelius pruinosus, Nylanderia fulva,* and *Pheidole obscurithorax* (Table S1)*.* The counts of the aforementioned species were negligible, thus excluded from the reported analyses.

**Figure 1:**
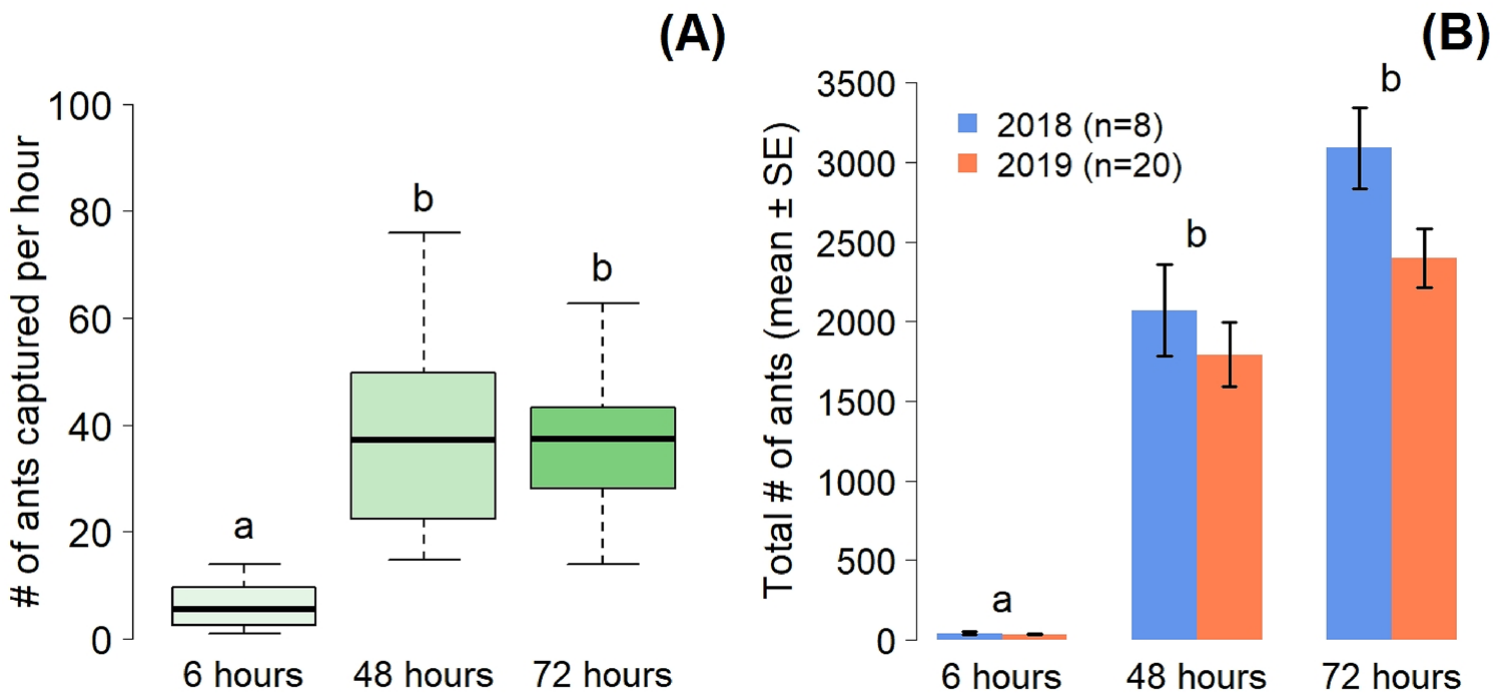
(**A**) Ant activity as ants captured per hour since carrion placement is obtained by the mean number of fire ants (from both 2018 and 2019) around each carcass was divided by the number of hours since rat carrion placement. (**B**) Mean number of ants per pair of pitfall traps placed adjacent to the rat carcass for the samples collected in 2018 (blue) and 2019 (red). Letters above bars represent the results of the post-hoc pairwise comparison analysis. Bars sharing a letter are not significantly different (P < 0.05).

The number of ants captured hourly in the pitfall traps surrounding the rat carrion (Fig. 1A) significantly increased throughout the experiment (GLMM: χ^2^ = 120.05; df = 2; *P* < 0.001). This increase was significant between 6 and 48 h (*post-hoc* pairwise comparisons: Z = 10.56; *P* < 0.001), with an average number of ants collected per hour rising from 6.2 ± 0.76 after 6 h to 39.00 ± 3.44 after 48 h. The ant activity remained high after 72 h with no significant difference detected between 48 and 72 h (Z = − 0.34; *P* = 1), with an average of 36.08 ± 2.22 ants captured per hour, 72 h after rat placement.

The total number of ants collected was significantly different between time points, but there were no significant differences between the two years of experimentation (GLM: Time: χ^2^ = 688.27; df = 2; *P* < 0.001. Year: χ^2^ = 2.54; df = 1; *P* = 0.111. Time:Year: χ^2^ = 0.16; df = 2; *P* = 0.921) (Fig. 1B). On average over the years 2018 and 2019, 38 ants were collected at 6 h, 1931 ants at 48 h and 2745 ants at 72 h (Fig. 1B).

### Laboratory colony performance experiment

**Figure 2:**
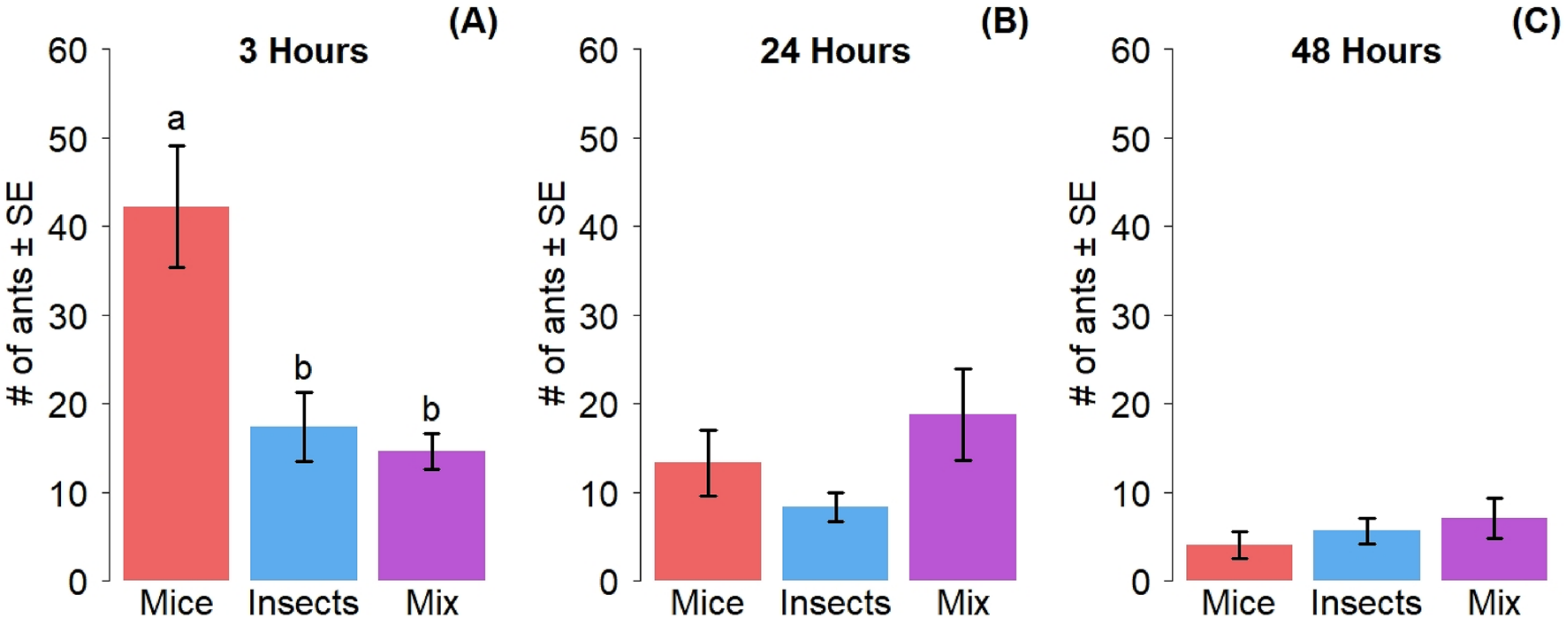
Number of ants counted in food dishes (mean ± SE) as a function of food treatments (**A**) at three hours (n = 6), (**B**) at 24 h (n = 6) and (**C**) at 48 h (n = 6). Letters above bars represent the results of the post-hoc pairwise comparison analysis, bars sharing a letter are not significantly different (P < 0.05). Red = mice, blue = insects, purple = mix.

Three hours after the replacement of food dishes, the number of ants counted in the dishes was significantly different depending on the treatment (GLMM: df = 2, χ^2^ = 102.99, *P < *0.001) (Fig. 2A). The recruitment to mice was significantly the highest, with an average of 42.21 ± 6.81 ants, followed by insects at 17.42 ± 3.92 ants and the mix of mice and insects at 14.67 ± 1.99 ants. At 24 h (Fig. 2B) and 48 h (Fig. 2C) after food replacement, no significant differences between food treatments were detected (GLMM: 24 h: df = 2, χ^2^ = 5.62, *P* = 0.060; 48 h: df = 2, χ^2^ = 3.60, *P* = 0.166).

**Figure 3:**
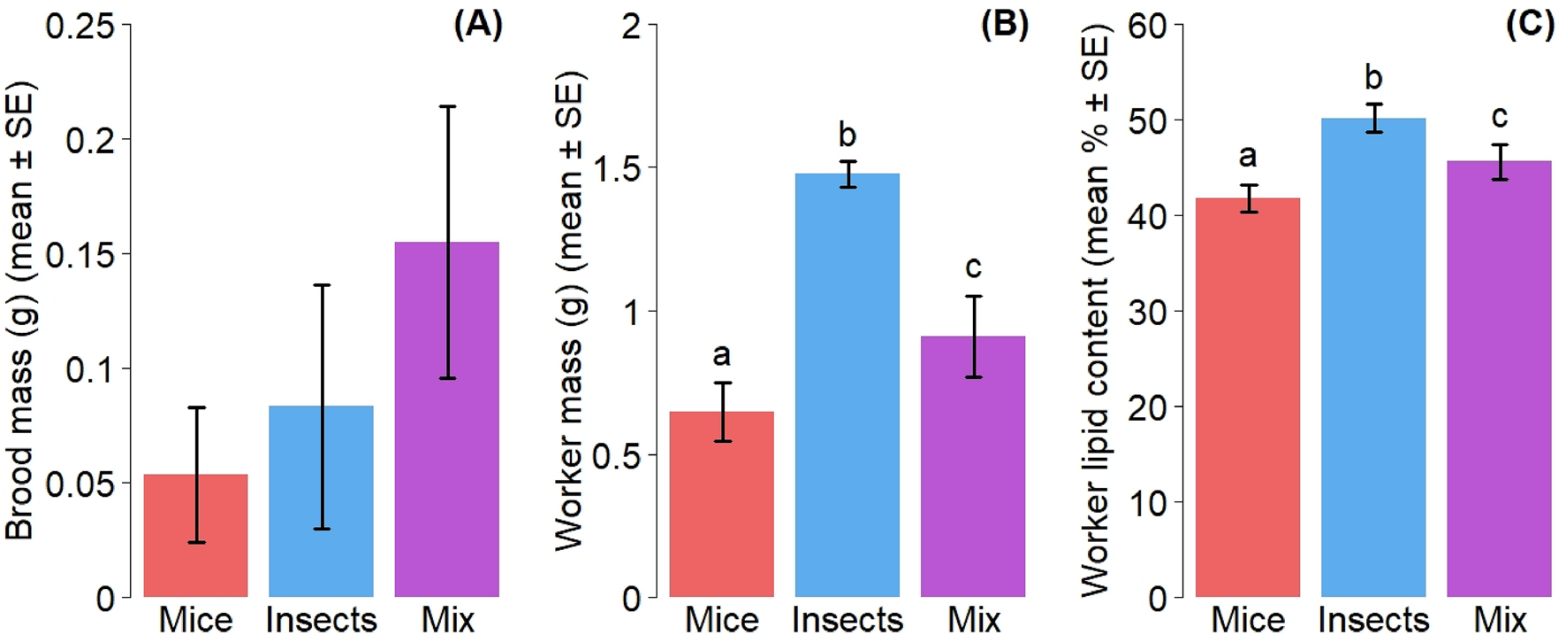
(**A**) Final brood mass of experimental colonies (mean ± SE), (**B**) final worker mass of experimental colonies (mean ± SE) and (**C**) proportion of lipids per ant (mean ± SE) as a functions of food treatments. Red = mice (n = 6), blue = insect (n = 5), purple = mix (n = 6).

Worker mass, an estimate of worker production, was significantly affected by diet treatments (GLMM: df = 2, χ^2^ = 64.95, *P* < 0.001) (Fig. 3B). Insect-fed colonies, on average, yielded the highest worker mass with 1.48 ± 0.046 g (mean ± SE), followed by mixed diet-fed colonies at 0.91 ± 0.14 and mice-fed colonies at 0.65 ± 0.10 g. Our results also indicated an influence of food treatment on the ant worker lipid content (GLMM: df = 2, χ^2^ = 19.52, *P* < 0.001) (Fig. 3C). Insect treatment was associated with the highest percentage of lipid per ant at 50 ± 1.49%, followed by mix treatment with 46 ± 1.78% and finally mice treatment with 42 ± 1.42%. The masses of brood collected from the laboratory colonies at the end of the experiment, however, were not significantly affected by the food treatment (GLMM: df = 2, χ^2^ = 2.57, *P* = 0.277) (Fig. 3A).

**Figure 4:**
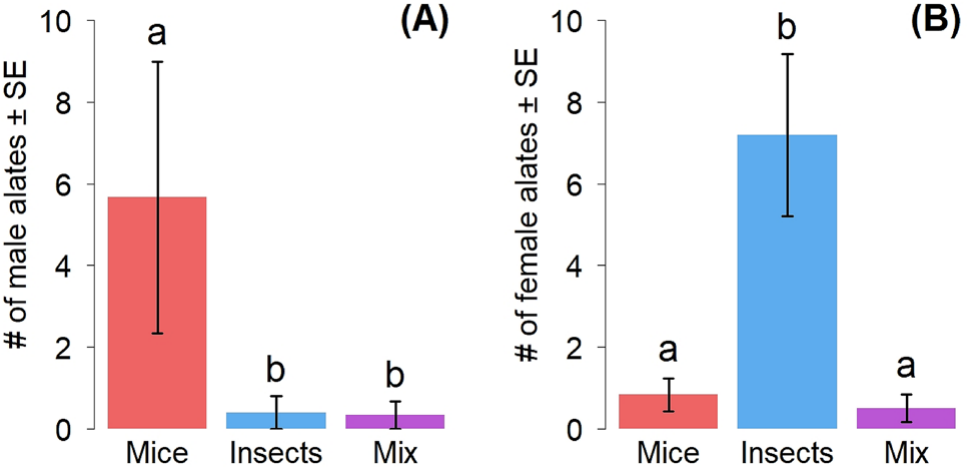
(**A**) Number of male alates counted in experimental colonies (mean ± SE) and (**B**) number of female alates counted in experimental colonies (mean ± SE) as functions of food treatments. Red = mice (n = 6), blue = insect (n = 5), purple = mix (n = 6). Letters above bars represent the results of the post-hoc pairwise comparison analysis. Bars sharing a letter are not significantly different (P < 0.05).

Finally, colonies fed with the mice diet had a significantly higher number of male alates (5.67 ± 3.32) compared to insect (0.40 ± 0.40) and mix (0.33 ± 0.33) diets (GLMM: df = 2, χ^2^ = 26.51, *P* < 0.001) (Fig. 4A). Colonies that were fed insect diets produced significantly more female alates (7.20 ± 1.98), exceeding the other treatments (mice: 0.83 ± 0.40; mix: 0.50 ± 0.34) (GLMM: df = 2, χ^2^ = 35.36, *P* < 0.001) (Fig. 4B).

## Discussion

This study shows that rat carcasses left out in the field are quickly and without exception colonized by red imported fire ants at high densities. This strong attraction of fire ants to rodent carrion was also observed under controlled, laboratory conditions: three hours post food treatment placement, mice attracted on average twice as many ants than did insect carrion. Interestingly, our analyses assessing ant colony performance suggested that vertebrate carrion is a lower-quality resource, alone or in combination with insect carrion. Our insect diet treatment composed of crickets and superworms resulted in significantly higher worker mass (an estimate of worker production) and worker lipid content than a diet composed of vertebrate carrion alone or supplemented with insects. Altogether, our results indicate that heavy fire ant recruitment to vertebrate carrion is likely not due to the nutritional benefits of ingesting vertebrate carrion. However, it is useful to note that the choices provided to the ants in this study were artificially limited, which could potentially impact interpretation. If there is a benefit to feeding on vertebrate remains, it may be a short-term benefit that can be detrimental in longer term exposures or with a lack of additional nutritional sources that can balance deleterious effects. Such an explanation could be considered a vertebrate carrion as “junk food” hypothesis.

The study of ant nutrition has mainly been driven by the need to rear ant colonies for laboratory experiments. Generally, diets for rearing fire ant laboratory colonies consists of dead insects, such as crickets and mealworms^39^. Earlier studies demonstrated that insects are imperative for normal larval growth in fire ant colonies^40–42^. The idea of substituting insects or supplementing fire ant diet with vertebrate carrion is not novel, as some have attempted to find a cheaper, more consistent and controlled alternative food for laboratory colonies^25,39,43,44^. Williams et al.^41^, for example, found that feeding fire ants raw or fried ground beef produced smaller, non-melanized neonates that weighed less and were incapable of stinging when compared to fire ants that were fed crickets. Though Gavilanez-Slone and Porter^39^ concluded that beef liver can substitute for insect prey when maximal colony growth rate is not necessary, the authors showed that house crickets (*Acheta domesticus*) still outperformed all types of liver diets (raw, boiled and mixed with agar, and raw and mixed with agar). Similarly, another study fed fire ants either raw beef liver, raw chicken liver or domestic crickets resulted in liver-fed colonies’ brood production collapse which may be related to missing micronutrients, unknown pathogens, the accumulation of toxins, or a combination of these and other unknown factors^44^. One obvious difference in these foods is the presence of chitin in insect-derived diets, which is a critical building block of insect exoskeletons that could explain better performance of ants, which also require chitin to produce an exoskeleton, on insect remains.

In our study, we show that fire ant colonies performed relatively poorly when fed dead mice, suggesting that vertebrate carrion does not provide rare nutrients that cannot be found in an insect-based diet; thus, the attraction to vertebrate carrion is likely not linked to the nutritional value. The protein and lipid content of mice is 61.2 and 25.9% of dry mass (dm), crickets have 62.0 and 33.3% of dm and superworms have 55.0 and 42.2% of dm, respectively (nutritional composition of mice provided by RodentPro.com; nutritional composition of crickets and superworms provided by Ghann’s Cricket Farm, Inc.). Nutritional studies identified the preferred nutritional intake for ants, focusing on the proportions of proteins and carbohydrates^26^. Fire ants, in particular, prefer a carbohydrate biased diet (between 14–21% protein and between 62–69% carbohydrate of dm)^34^. In the present study, our diet treatments were protein biased, thus probably lower quality for fire ants. Previous studies have shown that high protein diets can lead to premature death in the ant *Linepithema humile* due to the toxicity of certain amino acids^34,45^. The effect of lipid consumption on the performance of ant colonies, however, is not well-studied in ants (reviewed by Csata and Dussutour^26^). In mice, there is approximately 2.4 times more protein than there is lipid. This ratio is more balanced in crickets (1.9:1 P:L) as well as in superworms (1:3 P:L), making the insect diet treatment’s lower protein ratio nutritionally better for fire ants.

The production of reproductives by colonies also suggests that vertebrate carrion is inferior for fire ant nutrition. Fire ant colonies that were fed a diet of mice carrion only produced a significantly higher number of male alates, whereas significantly more females were found in the colonies that were fed only insects. Female alates are more costly to produce and maintain in comparison to their male counterparts^46^, further suggesting that insects are nutritionally richer than mice carrion. Colonies that are facing lower nutritional input typically invest in the production of male reproductives^47^, which may be a “last-ditch effort” for dispersal. Such nutritionally-based sex allocation has been found in multiple animals, including other species of ants^48,49^.

While a large body of evidence points towards the poor nutritional value of vertebrate carrion, numerous studies have reported ants on, around or feeding on vertebrate carrion^10,11^. From a proximate perspective, the attraction to resources is based on the detection of olfactory and gustatory cues that informs the recipient of its nutritional value and suitability for consumption and is well-documented in ants^50^. However, the present study suggests the existence of a possible discrepancy between the attractive cues and the nutritional value when it comes to vertebrate carrion. Olfactory cues emitted by vertebrate carrion (e.g., indole and dimethyl-disulfide^51,52^) have mostly been studied in a forensic entomology context, showing their role in the attraction of the common green bottle fly, *Lucilia sericata*^53^, and one of its parasites, the parasitoid wasp *Nasonia vitripennis*^54^. The common green bottle fly (*L. sericata)* uses these attractive cues to locate a food source (vertebrate carrion) and the parasitoid (*N. vitripennis*) uses these cues to locate a host (maggots) that is associated with vertebrate carrion. Studies of vertebrate carrion volatiles in the context of ant foraging behavior are still lacking but we hypothesize that a mechanism similar to the one identified in the *L. sericata*—*N. vitripenis* system is at play. In the same way that *N. vitripenis* is attracted to vertebrate carrion volatiles as a cue for the presence of *L. sericata* maggots (hosts), we predict that fire ants are attracted to vertebrate carrion volatiles as a cue for the presence of other carrion-visiting insects. Ant predation on carrion-visiting insects have been reported in several studies, as reviewed in Eubanks et al. (2019). Such indirect attraction involving ants exists in the context of tritrophic interactions; Schettino et al.^55^ showed that *Formica pratensis* workers are attracted to the volatiles emitted from cucumber and potato plants when the plants were attacked by aphids and caterpillars, where the plants recruit ants for protection and in return, ants predate on the herbivores. In essence, the vertebrate carrion, vertebrate carrion-visitor and fire ant system may be another instance of a tritrophic interaction. Recently, fire ants were recorded harassing female blow flies on rodent and poultry carcasses to such a degree that they elicited a wing buzzing phenotype in response to the ants^56^. Interestingly, the flies in that study persisted in searching for oviposition sites in the face of ant presence on a resource, suggesting that ant recruitment to these remains would ultimately result in encounters with easily predated eggs and larvae. Our study shows a deleterious effect of the introduction of mice carrion in fire ant diet, which supports our hypothesis stating that there may be indirect benefits associated with the colonization of vertebrate carrion (i.e., gaining access to a large population of vertebrate carrion-visiting insects to predate upon) that outweighs the costs of consuming it.

Here, we show that fire ants readily colonize vertebrate carrion in the field. Though many have drawn attention to the potential importance of vertebrate carrion in ant diets and the importance of ants on vertebrate carrion decomposition, the present study is one of the first to provide data on the relationship between fire ant colony performance and vertebrate carrion. We found that although fire ants recruit heavily to vertebrate carrion, it is likely that fire ants benefit more from predating carrion-feeding insects than feeding on vertebrate carrion, showcasing a tritrophic interaction. Future studies should directly quantify ant predation of vertebrate carrion-feeding insects, as such predators could play an important role in structuring the diversity and dynamics of these carrion communities (see Wells & Greenberg^57^ for indirect influence). Future studies could also compare the performance of colonies on vertebrate carrion and vertebrate carrion colonized by carrion-feeding insect larvae. Providing a broader range of nutritional choices to fire ants may help to dissect if vertebrate carrion serves a role as a “junk food” that needs to be balanced. Regardless of why, fire ants appear to demonstrate a preference for vertebrate carrion that outweighs its value as a resource.

## Methods

### Field experiment

This experiment was designed to quantify ant recruitment to vertebrate, specifically rodent, carrion. Field experiments were conducted at the Ecology and Natural Resource Teaching Area near Easterwood Airport in College Station, Texas, USA (30.576303, − 96.364108) in 2018 and 2019 between the months of May and September. Habitat type of this field site is an open post oak savannah with medium fire ant mound density. Vegetation included few large post oaks (*Quercus stellata*) and mixed grasses (ex. *Panicum virgatum*, *Sorghastrum nutans*, *Schizachyrium scoparium*, *Paspalum floridanum*, *Stapfochloa canterae*, *Croton lindheimeri*, *Eragrostis superba*, etc.). The field was sectioned into six plots (25 × 30 m). Plots were separated in all directions by at least 25 m. It is important to note that there were no large trees that cast shade on any of the experimental plots. Plots were randomly assigned to one of three lengths of decomposition (6, 48 or 72 h) to assess recruitment over time. Beginning on day one of the experiment, one brown rat (*Rattus norvegicus*) carcass (weighing between 375.00 – 475.00 g) (commercially-sourced from RodentPro.com), was placed in the center of each plot along with a small pitfall trap (50 mL centrifuge tube) and a large pitfall trap (266.162 mL plastic cup) both filled with filled with a solution of unscented soapy water and salt. Small pitfall traps were to capture the smaller target insects (e.g., ants) and the large pitfall traps were to capture larger invertebrate visitors (e.g., carrion beetles). A 1m^2^ cage built of wire mesh (1 in. hexagonal mesh size) and steel mesh sheet (4 in. × 6 in. spacing) was placed over the rat carcass to exclude vertebrate scavengers. Carcasses were placed at least 25 m apart. Vertebrate carrion was left to decompose for different lengths of time (6, 48 and 72 h) and data collection occurred at the end of each time interval. Data collection at each time interval consisted of: (1) visual inspection of the carcass to determine ant colonization (2) pitfall trap collection and (3) destructive sampling of carcasses. All specimens of insects collected from the field were preserved in 70% ethanol then later counted and identified. Eight replications were conducted in 2018 and 20 in 2019.

#### Data analysis

The number of fire ants around each carcass was divided by the number of hours since carrion placement to obtain a measure of ant activity expressed in ants captured per hour since carrion placement. Raw ant count data was not used as it is certain that there should be more ants captured the more time has passed.

Number of ants captured in pitfall traps was compared between times elapsed since carrion placement (6, 48 and 72 h) using a general linear mixed effect model in the R package *glmmTMB* with a negative binomial distribution (since ant activity is based on count data)^29^. Number of ants was the dependent variable; time was the fixed independent variable and year was considered a random effect. Model diagnostics were computed using the R package *DHARMa*^30^. Results of the model are presented as an ANOVA table using the *Anova* function from the R package *car*^31^ using type II Wald χ^2^ test. *Post-hoc* pairwise comparisons were computed with the R package *multcomp*^32^ and *P-*values were adjusted with the false discovery rate correction (FDR). Unless specifically reported otherwise, all values are given as mean ± SE of mean.

### Laboratory experiment

#### Colony collection and maintenance:

Six fire ant colonies were collected in the field around College Station, Texas during the month of June in 2019. Across Texas, polygyne fire ant colonies occur more frequently than monogyne colonies^33^. Fire ants collected for this experiment were all polygyne (personal confirmation). Three standardized experimental colonies consisting of one queen, ~ 0.5 g brood (~ 1000 larvae) and ~ 1.5 g wet mass of workers (~ 3000 ants) each (yielding 18 experimental colonies). To ensure that all experimental colonies are in the similar nutritional states, experimental colonies were maintained in individual colony habitats for a week under standardized laboratory conditions (temperature 24–32 °C, 40–70% humidity, 12:12 light/dark cycle) and fed diets of freshly killed adult crickets (*Gryllodes sigillatus*), superworms (*Zophobas morio* larvae) (both acquired from Ghann’s Cricket Farms, Inc.), water and 10% honey water, ad libitum.

#### Experiment

All colonies were starved for 24 h prior to the beginning of the experiment. The three experimental colonies from the same field colony of origin were randomly assigned to one of the following diet treatments: “insect”, “mixed” and “mice”; with “insect” representing colonies that were fed ~ 6.0 g of insects (crickets and superworms carrion; 3 g each). “Mixed” representing colonies that were fed ~ 3.0 g hairless large pinky mice (weighing between 2.50–2.99 g) (RodentPro.com) and ~ 3.0 g dead insects. Lastly, “mice” representing ants that were fed ~ 6.0 g of hairless large pinky mice. All experimental colonies were supplemented with 10% honey water and water ad libitum. Ants were allowed to feed on the diet treatment for three days, which then was replaced by new food. Throughout the entirety of the experiment, the number of ants foraging on each treatment was visually assessed and counted at 3 h, 24 h, and 48 h after food (i.e., treatment) replacement. We counted ants to be foraging when the worker was inside the food dishes that contained the treatments. The experiment ran for 60 days (from June 27th, 2019, to August 26th, 2019), after which, all experimental colonies were placed into the freezer at − 20 °C for at least three days and then sorted into brood, workers and alates. At the end of the experiment, the final weight of brood and workers were taken, the number of workers were counted, and the queens were removed.

To estimate the lipid stores of the ants, we used the lipid extraction protocol modified after Cook et al.^34^. Five randomly selected workers were taken from each colony (three replicates; total of 15 ants were removed from each experimental colony), freeze dried for 24 h, then weighed to the nearest 0.01 mg using a microbalance to attain the dry mass. Dried specimens were placed into 2 ml Eppendorf vials along with 1 ml of chloroform, for 24 h. The chloroform was then pipetted out of the vial and discarded. Another 1 ml of chloroform was added back into the Eppendorf vials containing the ants and left for another 24 h then discarded. Samples were freeze dried again for 24 h and weighed to achieve the final lean mass. The Proportion of lipid was estimated using the following equation: ((dry mass – lean mass)/dry mass)^35,36^, then averaged per experimental colony.

#### Data analysis

The number of ants on the food dishes at each time point were analyzed separately (6, 48 and 72 h after food placement). For each time point, the number of ants counted on the food (dependent variable) was compared across food treatments (independent fixed variable) using a generalized linear mixed model with a negative binomial distribution (due to count data). The identity of the experimental colony was nested in natal colony and entered in the model as random effect to control for potential variation among natal colonies^37^. Results of the models are presented as ANOVA tables using the *Anova* function from the R package *car*^31^ using type II Wald χ^2^ test. Rat carcasses used in the field experiment were never sampled multiple times due to destructive sampling, thus a repeated measures analysis was inappropriate.

Final worker mass and final brood mass (dependent variable) were compared across food treatments (independent variable) using separate linear mixed models (LMM) with natal colony as a random effect. The proportion of lipids in workers was also compared across food treatments using an LMM. Values of the proportion of lipids aggregated around 0.5, which made using the gaussian distribution applicable. In this model, the random effect was the replicate nested within natal colony identity. Male and female alate counts were compared across food treatments using separate generalized linear mix models (GLMMs) with negative binomial distributions and natal colony identity as the random effect. All statistical models were computed using the *lme4* R package^38^ and the diagnostics using the package *DHARMa*^30^. Due to the death of a queen in the early stages of the experiment, one replicate of the insect diet treatment was removed, creating an imbalanced design, thus a type III error was assumed in the computation of the ANOVA table.

Post-hoc pairwise comparisons were computed using the package *multcomp*^32^ and the *P*-values were adjusted using the false discovery rate correction (FDR). Unless specifically reported otherwise, all values are given as mean ± SE of mean.

## Supplementary Information

Supplementary Information.
