# Supplementary material for: The discrepancy between fire ant recruitment to and performance on rodent carrion
Source: Sci Rep. 2022 Jan 7;12:71. doi: 10.1038/s41598-021-04051-8 (PMC8742042; doi:10.1038/s41598-021-04051-8)
Supplement: Supplementary file 1 — Supplementary Information. [file 41598_2021_4051_MOESM1_ESM.docx]

| **Ant Species** | **Count** | |
| --- | --- | --- |
|  | **2018 (n=8)** | **2019 (n=20)** |
| *Brachymyrmex patagonicus* | 75 (0.17%) | 242 (0.24%) |
| *Cyphomyrmex rimosus* | 3 (< 0.01%) | 0 (0%) |
| *Forelius pruinosus* | 1621 (3.74%) | 2937 (2.86%) |
| *Nylanderia fulva* | 20 (< 0.01%) | 11 (< 0.01%) |
| *Pheidole obscurithorax* | 16 (< 0.01%) | 89 (< 0.01%) |
| *Solenopsis invicta* | 41620 (96.00%) | 99312 (96.80%) |

**Table S1.** Counts of all ant species captured in pitfall traps. All time points were combined within each year with the proportion (reported in percentages) of each species shown between parentheses.
